# *N*-(2,4-Dichloro­phen­yl)-4-methyl­benzene­sulfonamide

**DOI:** 10.1107/S1600536811030819

**Published:** 2011-08-06

**Authors:** K. Shakuntala, Sabine Foro, B. Thimme Gowda

**Affiliations:** a Department of Chemistry, Mangalore University, Mangalagangotri 574 199, Mangalore, India; b Institute of Materials Science, Darmstadt University of Technology, Petersenstrasse 23, D-64287 Darmstadt, Germany

## Abstract

The mol­ecule of the title compound, C_13_H_11_Cl_2_NO_2_S, is bent at the S atom with a C—SO_2_—NH—C torsion angle of −69.07 (16)°. The sulfonyl and aniline rings are rotated relative to each other by 53.0 (1)°. In the crystal, pairs of N—H⋯O(S) hydrogen bonds link the mol­ecules into centrosymmetric dimers.

## Related literature

For hydrogen-bonding modes of sulfonamides, see: Adsmond & Grant (2001 ▶). For studies on the effects of substituents on the structures and other aspects of *N*-(ar­yl)-amides, see: Arjunan *et al.* (2004 ▶); Gowda *et al.* (2000 ▶, 2006 ▶), on *N*-(ar­yl)-methane­sulfonamides, see: Gowda *et al.* (2007 ▶) and on *N*-(ar­yl)-aryl­sulfonamides, see: Gelbrich *et al.* (2007 ▶); Perlovich *et al.* (2006 ▶); Shakuntala *et al.* (2010 ▶). For the preparation of the title compound, see: Shetty & Gowda (2005 ▶)
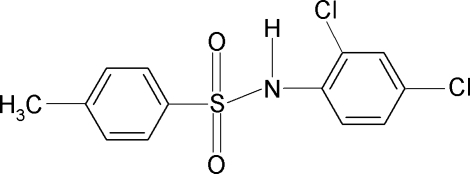


## Experimental

### 

#### Crystal data

C_13_H_11_Cl_2_NO_2_S
*M* *_r_* = 316.19
Monoclinic, 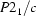

*a* = 11.385 (1) Å
*b* = 11.959 (1) Å
*c* = 11.428 (1) Å
β = 112.87 (2)°
*V* = 1433.6 (2) Å^3^
*Z* = 4
Mo *K*α radiation
μ = 0.59 mm^−1^
*T* = 293 K
0.44 × 0.38 × 0.28 mm

#### Data collection

Oxford Diffraction Xcalibur diffractometer with a Sapphire CCD detector
Absorption correction: multi-scan (*CrysAlis RED*; Oxford Diffraction, 2009 ▶) *T*_min_ = 0.780, *T*_max_ = 0.851
5752 measured reflections
2924 independent reflections
2361 reflections with *I* > 2σ(*I*)
*R*_int_ = 0.013

#### Refinement

*R*[*F* ^2^ > 2σ(*F* ^2^)] = 0.036
*wR*(*F* ^2^) = 0.103
*S* = 1.04
2924 reflections
177 parameters
1 restraint
H atoms treated by a mixture of independent and constrained refinement
Δρ_max_ = 0.28 e Å^−3^
Δρ_min_ = −0.38 e Å^−3^

### 

Data collection: *CrysAlis CCD* (Oxford Diffraction, 2009 ▶); cell refinement: *CrysAlis RED* (Oxford Diffraction, 2009 ▶); data reduction: *CrysAlis RED*; program(s) used to solve structure: *SHELXS97* (Sheldrick, 2008 ▶); program(s) used to refine structure: *SHELXL97* (Sheldrick, 2008 ▶); molecular graphics: *PLATON* (Spek, 2009 ▶); software used to prepare material for publication: *SHELXL97*.

## Supplementary Material

Crystal structure: contains datablock(s) I, global. DOI: 10.1107/S1600536811030819/bt5590sup1.cif

Structure factors: contains datablock(s) I. DOI: 10.1107/S1600536811030819/bt5590Isup2.hkl

Supplementary material file. DOI: 10.1107/S1600536811030819/bt5590Isup3.cml

Additional supplementary materials:  crystallographic information; 3D view; checkCIF report

## Figures and Tables

**Table 1:** Hydrogen-bond geometry (Å, °)

*D*—H⋯*A*	*D*—H	H⋯*A*	*D*⋯*A*	*D*—H⋯*A*
N1—H1*N*⋯O1^i^	0.85 (2)	2.21 (2)	3.024 (2)	160 (2)

Symmetry code: (i) 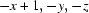.

**Fig. 1.**
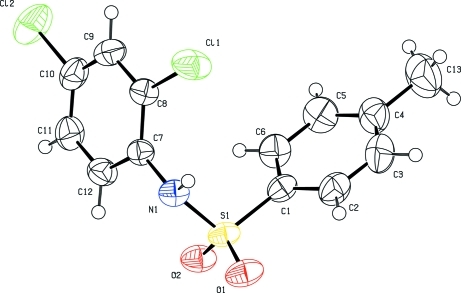
Molecular structure of the title compound, showing the atom labelling scheme and displacement ellipsoids are drawn at the 50% probability level.

**Fig. 2.**
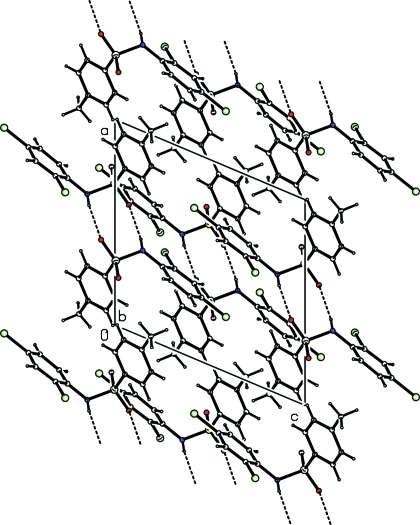
Molecular packing of (I) with hydrogen bonding shown as dashed lines.

## supplementary crystallographic information

### Comment

The amide and sulfonamide moieties are the constituents of many biologically significant compounds. The hydrogen bonding preferences of sulfonamides have been investigated (Adsmond & Grant, 2001). As part of our work on the substituent effects on the structures and other aspects of *N*-(aryl)-amides (Arjunan *et al.*, 2004; Gowda *et al.*, 2000, 2006), *N*-(aryl)-methanesulfonamides (Gowda *et al.*, 2007) and *N*-(aryl)-arylsulfonamides (Shakuntala *et al.*, 2010), in the present work, the crystal structure of *N*-(2,4-dichlorophenyl)- 4-methylbenzenesulfonamide (I) has been determined (Fig. 1).

The conformation of the N—H bond in the C—SO_2_—NH—C segment and the *ortho*-chloro group in the anilino benzene ring are *syn* to each other. The molecule is bent at the S atom with the C—SO_2_—NH—C torsion angle of -69.1 (2)°, compared to the values of 65.4 (2) and -61.7 (2)° in the two molecules of *N*-(2,3-dichlorophenyl)- 4-methylbenzenesulfonamide (II)(Shakuntala *et al.*, 2010).

The sulfonyl and the aniline benzene rings in (I) are tilted relative to each other by 53.0 (1)°, compared to the values of 76.0 (1) and 79.9 (1)° in the two molecules of (II). The other bond parameters in (I) are similar to those observed in (II) and other aryl sulfonamides (Perlovich *et al.*, 2006; Gelbrich *et al.*, 2007).

In the crystal, the N—H···O hydrogen bonds (Table 1) pack the molecules into zigzag chains in the direction parallel to *b*-axis (Fig. 2).

### Experimental

The solution of toluene (10 ml) in chloroform (40 ml) was treated dropwise with chlorosulfonic acid (25 ml) at 0 ° C. After the initial evolution of hydrogen chloride subsided, the reaction mixture was brought to room temperature and poured into crushed ice in a beaker. The chloroform layer was separated, washed with cold water and allowed to evaporate slowly. The residual 4-methylbenzenesulfonylchloride was treated with 2,4-dichloroaniline in the stoichiometric ratio and boiled for ten minutes. The reaction mixture was then cooled to room temperature and added to ice cold water (100 ml). The resultant *N*-(2,4-dichlorophenyl)-4-methylbenzenesulfonamide was filtered under suction and washed thoroughly with cold water. It was then recrystallized to constant melting point from dilute ethanol. The purity of the compound was checked and characterized by recording its infrared and NMR spectra (Shetty & Gowda, 2005).

The prism like colourless single crystals used in X-ray diffraction studies were grown in ethanolic solution by a slow evaporation at room temperature.

### Refinement

The H atom  of the NH group  was located in a difference map and later restrained to N—H = 0.86 (2) %A. The other H atoms were positioned with idealized geometry using a riding model with the aromatic C—H = 0.93Å and methyl C—H = 0.96 Å. The *U*_iso_(H) values were set at 1.2*U*_eq_(C-aromatic, N) and 1.5*U*_eq_(C-methyl).

### Crystal data

**Table:**

C_13_H_11_Cl_2_NO_2_S	*F*(000) = 648
*M**_r_* = 316.19	*D*_x_ = 1.465 Mg m^−^^3^
Monoclinic, *P*2_1_/*c*	Mo *K*α radiation, λ = 0.71073 Å
Hall symbol: -P 2ybc	Cell parameters from 2440 reflections
*a* = 11.385 (1) Å	θ = 2.6–27.7°
*b* = 11.959 (1) Å	µ = 0.59 mm^−^^1^
*c* = 11.428 (1) Å	*T* = 293 K
β = 112.87 (2)°	Prism, colourless
*V* = 1433.6 (2) Å^3^	0.44 × 0.38 × 0.28 mm
*Z* = 4	

### Data collection

**Table:**

Oxford Diffraction Xcalibur diffractometer with a Sapphire CCD detector	2924 independent reflections
Radiation source: fine-focus sealed tube	2361 reflections with *I* > 2σ(*I*)
graphite	*R*_int_ = 0.013
Rotation method data acquisition using ω and φ scans	θ_max_ = 26.4°, θ_min_ = 2.6°
Absorption correction: multi-scan (*CrysAlis RED*; Oxford Diffraction, 2009)	*h* = −14→14
*T*_min_ = 0.780, *T*_max_ = 0.851	*k* = −10→14
5752 measured reflections	*l* = −14→11

### Refinement

**Table:**

Refinement on *F*^2^	Secondary atom site location: difference Fourier map
Least-squares matrix: full	Hydrogen site location: inferred from neighbouring sites
*R*[*F*^2^ > 2σ(*F*^2^)] = 0.036	H atoms treated by a mixture of independent and constrained refinement
*wR*(*F*^2^) = 0.103	*w* = 1/[σ^2^(*F*_o_^2^) + (0.0569*P*)^2^ + 0.3942*P*] where *P* = (*F*_o_^2^ + 2*F*_c_^2^)/3
*S* = 1.04	(Δ/σ)_max_ = 0.018
2924 reflections	Δρ_max_ = 0.28 e Å^−^^3^
177 parameters	Δρ_min_ = −0.38 e Å^−^^3^
1 restraint	Extinction correction: *SHELXL97* (Sheldrick, 2008), Fc^*^=kFc[1+0.001xFc^2^λ^3^/sin(2θ)]^-1/4^
Primary atom site location: structure-invariant direct methods	Extinction coefficient: 0.070 (3)

### Special details

**Table:**

Geometry. All e.s.d.'s (except the e.s.d. in the dihedral angle between two l.s. planes) are estimated using the full covariance matrix. The cell e.s.d.'s are taken into account individually in the estimation of e.s.d.'s in distances, angles and torsion angles; correlations between e.s.d.'s in cell parameters are only used when they are defined by crystal symmetry. An approximate (isotropic) treatment of cell e.s.d.'s is used for estimating e.s.d.'s involving l.s. planes.
Refinement. Refinement of *F*^2^ against ALL reflections. The weighted *R*-factor *wR* and goodness of fit *S* are based on *F*^2^, conventional *R*-factors *R* are based on *F*, with *F* set to zero for negative *F*^2^. The threshold expression of *F*^2^ > σ(*F*^2^) is used only for calculating *R*-factors(gt) *etc*. and is not relevant to the choice of reflections for refinement. *R*-factors based on *F*^2^ are statistically about twice as large as those based on *F*, and *R*- factors based on ALL data will be even larger.

### Fractional atomic coordinates and isotropic or equivalent isotropic displacement parameters (Å^2^)

**Table:**

	*x*	*y*	*z*	*U*_iso_*/*U*_eq_	
C1	0.21863 (18)	−0.05135 (17)	−0.05214 (18)	0.0414 (4)	
C2	0.2305 (2)	−0.1166 (2)	−0.1463 (2)	0.0564 (6)	
H2	0.2911	−0.0991	−0.1794	0.068*	
C3	0.1525 (2)	−0.2075 (2)	−0.1910 (2)	0.0660 (6)	
H3	0.1595	−0.2500	−0.2561	0.079*	
C4	0.0635 (2)	−0.2378 (2)	−0.1417 (2)	0.0607 (6)	
C5	0.0538 (2)	−0.1719 (2)	−0.0458 (2)	0.0617 (6)	
H5	−0.0051	−0.1907	−0.0111	0.074*	
C6	0.1300 (2)	−0.07897 (19)	−0.0012 (2)	0.0525 (5)	
H6	0.1220	−0.0352	0.0626	0.063*	
C7	0.38204 (17)	0.02375 (15)	0.24980 (17)	0.0369 (4)	
C8	0.39658 (18)	−0.07796 (15)	0.31288 (18)	0.0418 (4)	
C9	0.3646 (2)	−0.09025 (18)	0.4174 (2)	0.0502 (5)	
H9	0.3785	−0.1577	0.4612	0.060*	
C10	0.3118 (2)	−0.0006 (2)	0.45504 (19)	0.0522 (5)	
C11	0.2956 (2)	0.10096 (19)	0.3946 (2)	0.0536 (5)	
H11	0.2600	0.1608	0.4212	0.064*	
C12	0.3327 (2)	0.11299 (17)	0.29395 (19)	0.0469 (5)	
H12	0.3245	0.1824	0.2548	0.056*	
C13	−0.0193 (3)	−0.3395 (3)	−0.1891 (3)	0.0923 (10)	
H13A	0.0322	−0.4014	−0.1944	0.111*	
H13B	−0.0835	−0.3245	−0.2717	0.111*	
H13C	−0.0593	−0.3577	−0.1315	0.111*	
N1	0.41946 (15)	0.03784 (14)	0.14553 (15)	0.0404 (4)	
H1N	0.4704 (18)	−0.0102 (15)	0.136 (2)	0.048*	
O1	0.38666 (14)	0.08540 (13)	−0.07316 (14)	0.0563 (4)	
O2	0.23685 (14)	0.15510 (12)	0.01841 (14)	0.0510 (4)	
Cl1	0.45623 (6)	−0.19221 (5)	0.26176 (6)	0.0665 (2)	
Cl2	0.26736 (7)	−0.01719 (8)	0.58308 (6)	0.0843 (3)	
S1	0.31493 (4)	0.06739 (4)	0.00383 (5)	0.04119 (17)	

### Atomic displacement parameters (Å^2^)

**Table:**

	*U*^11^	*U*^22^	*U*^33^	*U*^12^	*U*^13^	*U*^23^
C1	0.0392 (9)	0.0493 (11)	0.0380 (9)	0.0077 (8)	0.0176 (8)	0.0066 (8)
C2	0.0641 (13)	0.0662 (14)	0.0471 (11)	0.0042 (12)	0.0307 (11)	−0.0004 (11)
C3	0.0703 (16)	0.0719 (16)	0.0534 (13)	0.0024 (13)	0.0213 (12)	−0.0156 (12)
C4	0.0484 (12)	0.0623 (14)	0.0561 (13)	0.0007 (11)	0.0035 (10)	−0.0041 (11)
C5	0.0430 (11)	0.0757 (16)	0.0679 (14)	−0.0083 (11)	0.0231 (11)	−0.0012 (13)
C6	0.0468 (11)	0.0630 (14)	0.0561 (12)	−0.0011 (10)	0.0291 (10)	−0.0048 (10)
C7	0.0346 (9)	0.0384 (9)	0.0394 (9)	−0.0021 (8)	0.0162 (8)	0.0008 (8)
C8	0.0428 (10)	0.0384 (10)	0.0455 (10)	0.0012 (8)	0.0188 (8)	0.0020 (8)
C9	0.0547 (12)	0.0511 (12)	0.0464 (11)	−0.0006 (10)	0.0215 (10)	0.0104 (9)
C10	0.0545 (12)	0.0675 (14)	0.0395 (10)	−0.0056 (11)	0.0239 (9)	−0.0030 (10)
C11	0.0616 (13)	0.0539 (12)	0.0516 (12)	0.0034 (11)	0.0287 (11)	−0.0109 (10)
C12	0.0559 (12)	0.0376 (10)	0.0518 (11)	0.0019 (9)	0.0259 (10)	0.0000 (9)
C13	0.0752 (19)	0.086 (2)	0.095 (2)	−0.0204 (17)	0.0106 (16)	−0.0174 (17)
N1	0.0374 (8)	0.0428 (9)	0.0468 (9)	0.0039 (7)	0.0227 (7)	0.0048 (7)
O1	0.0604 (9)	0.0663 (10)	0.0574 (9)	0.0063 (8)	0.0395 (8)	0.0187 (7)
O2	0.0544 (8)	0.0441 (8)	0.0608 (9)	0.0134 (7)	0.0294 (7)	0.0134 (7)
Cl1	0.0877 (5)	0.0423 (3)	0.0857 (4)	0.0176 (3)	0.0513 (4)	0.0088 (3)
Cl2	0.0955 (5)	0.1192 (6)	0.0568 (4)	−0.0017 (5)	0.0500 (4)	0.0039 (4)
S1	0.0430 (3)	0.0440 (3)	0.0447 (3)	0.0062 (2)	0.0259 (2)	0.0116 (2)

### Geometric parameters (Å, °)

**Table:**

C1—C2	1.378 (3)	C8—Cl1	1.7256 (19)
C1—C6	1.387 (3)	C9—C10	1.377 (3)
C1—S1	1.756 (2)	C9—H9	0.9300
C2—C3	1.371 (3)	C10—C11	1.374 (3)
C2—H2	0.9300	C10—Cl2	1.736 (2)
C3—C4	1.385 (3)	C11—C12	1.378 (3)
C3—H3	0.9300	C11—H11	0.9300
C4—C5	1.389 (3)	C12—H12	0.9300
C4—C13	1.505 (4)	C13—H13A	0.9600
C5—C6	1.380 (3)	C13—H13B	0.9600
C5—H5	0.9300	C13—H13C	0.9600
C6—H6	0.9300	N1—S1	1.6326 (17)
C7—C12	1.389 (3)	N1—H1N	0.852 (15)
C7—C8	1.391 (3)	O1—S1	1.4304 (13)
C7—N1	1.423 (2)	O2—S1	1.4260 (14)
C8—C9	1.385 (3)		
			
C2—C1—C6	120.1 (2)	C8—C9—H9	120.7
C2—C1—S1	120.38 (16)	C11—C10—C9	121.39 (19)
C6—C1—S1	119.53 (16)	C11—C10—Cl2	119.92 (17)
C3—C2—C1	119.6 (2)	C9—C10—Cl2	118.68 (17)
C3—C2—H2	120.2	C10—C11—C12	119.14 (19)
C1—C2—H2	120.2	C10—C11—H11	120.4
C2—C3—C4	121.8 (2)	C12—C11—H11	120.4
C2—C3—H3	119.1	C11—C12—C7	121.42 (19)
C4—C3—H3	119.1	C11—C12—H12	119.3
C3—C4—C5	117.9 (2)	C7—C12—H12	119.3
C3—C4—C13	121.3 (2)	C4—C13—H13A	109.5
C5—C4—C13	120.8 (2)	C4—C13—H13B	109.5
C6—C5—C4	121.2 (2)	H13A—C13—H13B	109.5
C6—C5—H5	119.4	C4—C13—H13C	109.5
C4—C5—H5	119.4	H13A—C13—H13C	109.5
C5—C6—C1	119.5 (2)	H13B—C13—H13C	109.5
C5—C6—H6	120.3	C7—N1—S1	121.08 (12)
C1—C6—H6	120.3	C7—N1—H1N	117.6 (15)
C12—C7—C8	117.85 (17)	S1—N1—H1N	107.0 (15)
C12—C7—N1	120.55 (16)	O2—S1—O1	119.47 (9)
C8—C7—N1	121.58 (16)	O2—S1—N1	106.89 (9)
C9—C8—C7	121.44 (18)	O1—S1—N1	105.80 (9)
C9—C8—Cl1	118.54 (15)	O2—S1—C1	107.95 (9)
C7—C8—Cl1	120.02 (14)	O1—S1—C1	108.83 (9)
C10—C9—C8	118.67 (19)	N1—S1—C1	107.31 (9)
C10—C9—H9	120.7		
			
C6—C1—C2—C3	1.3 (3)	C8—C9—C10—Cl2	178.11 (16)
S1—C1—C2—C3	−178.49 (18)	C9—C10—C11—C12	0.1 (3)
C1—C2—C3—C4	−1.7 (4)	Cl2—C10—C11—C12	179.30 (17)
C2—C3—C4—C5	0.9 (4)	C10—C11—C12—C7	2.2 (3)
C2—C3—C4—C13	−178.4 (2)	C8—C7—C12—C11	−1.9 (3)
C3—C4—C5—C6	0.2 (4)	N1—C7—C12—C11	179.82 (18)
C13—C4—C5—C6	179.5 (2)	C12—C7—N1—S1	−65.1 (2)
C4—C5—C6—C1	−0.6 (3)	C8—C7—N1—S1	116.64 (18)
C2—C1—C6—C5	−0.2 (3)	C7—N1—S1—O2	46.53 (16)
S1—C1—C6—C5	179.58 (17)	C7—N1—S1—O1	174.86 (14)
C12—C7—C8—C9	−0.8 (3)	C7—N1—S1—C1	−69.07 (16)
N1—C7—C8—C9	177.50 (18)	C2—C1—S1—O2	137.68 (17)
C12—C7—C8—Cl1	179.49 (15)	C6—C1—S1—O2	−42.10 (18)
N1—C7—C8—Cl1	−2.2 (3)	C2—C1—S1—O1	6.6 (2)
C7—C8—C9—C10	3.0 (3)	C6—C1—S1—O1	−173.16 (16)
Cl1—C8—C9—C10	−177.23 (17)	C2—C1—S1—N1	−107.43 (17)
C8—C9—C10—C11	−2.7 (3)	C6—C1—S1—N1	72.78 (18)

### Hydrogen-bond geometry (Å, °)

**Table:**

*D*—H···*A*	*D*—H	H···*A*	*D*···*A*	*D*—H···*A*
N1—H1N···O1^i^	0.85 (2)	2.21 (2)	3.024 (2)	160.(2)

Symmetry codes: (i) −*x*+1, −*y*, −*z*.
